# Centering Patient and Clinician Voices in Developing Tools to Address Pain Related School Impairment: A Phase I Study of a Virtual Reality School Simulation for Children and Adolescents with Chronic Pain

**DOI:** 10.3390/children10101644

**Published:** 2023-10-01

**Authors:** Deirdre E. Logan, Karina Khanna, Edin Randall, Shealyn O’Donnell, Talis Reks, Logan McLennan

**Affiliations:** 1 Department of Psychiatry, Harvard Medical School, Boston, MA 02115, USA; 2 Department of Anesthesiology, Critical Care and Pain Medicine, Boston Children’s Hospital, Boston, MA 02115, USA; 3 Mayo Family Pediatric Pain Rehabilitation Center, Boston Children’s Hospital, Boston, MA 02115, USA; 4 Massachusetts Institute of Technology, Cambridge, MA 02139, USA; 5 Tufts University School of Arts and Sciences, Medford, MA 02153, USA; lrmclennan01@gmail.com

**Keywords:** virtual reality, pediatric pain, school functioning, digital health

## Abstract

Building on growing evidence supporting virtual reality (VR) interventions for pain management, this study describes the process of developing vReal-School (vRS), a VR-based school simulation for children and adolescents with chronic pain and associated school impairment. Following guidelines for developing user-centered VR interventions, initial phases of intervention development focus on understanding and incorporating patient and clinician perspectives when designing this digital health tool. Phase I entailed focus groups with patients undergoing intensive interdisciplinary pain treatment (IIPT). A total of 19 participants across four focus groups shared their experiences related to dealing with pain at school and provided initial feedback on the concept of a VR-based school simulation. In phase II, we pilot-tested a vRS prototype and collected patient and clinician feedback via mixed method approaches. Phase I results highlight four themes related to pain in school, including physical/environmental challenges and solutions, academic challenges and solutions, peer interaction challenges and solutions, and teacher interaction challenges and solutions. These themes guided the development of our vRS prototype. Nine patients and eleven treating clinicians then engaged with the vRS prototype and provided feedback via semi-structured interviews and validated self-report measures. The results indicate high levels of patient engagement/immersion (mean total score of 17.0 on the Child Presence Measure). Qualitative feedback from both groups identified positive aspects of vRS, including finding the simulation realistic and easy to use and offering ways to address school functioning goals that are not otherwise feasible in the IIPT setting. Areas for improvement included integrating more physical movement as well as increasing the number of scenarios and the level of demands of the tasks available. Both patients and clinicians found vRS to be useful in the IIPT context and relevant to treatment goals. This user input will guide subsequent iterations of intervention development.

## 1. Introduction

The integration of digital health technologies into the treatment of acute and chronic pain has accelerated in the last decade, offering new, safe alternative treatment approaches to pain management and reducing the need to rely on medication-based pain treatment [1,2,3,4]. Digital health tools have the potential to increase patient engagement, reduce access barriers, and enhance patient-centered care, with the central goal of alleviating pain and disability in patients with chronic pain. Within the realm of digital health innovations, virtual reality (VR) is an effective tool that allows users to engage in interactive computer-generated environments, harnessing multiple sensory inputs to provide an immersive mind–body experience that can facilitate therapeutic goals. Children and adolescents are particularly well-suited to benefit from VR pain interventions given their facility with technology and the ease with which they engage in imaginative experiences [2,5,6].

To address chronic pain, VR interventions must provide immersive experiences that go beyond redirecting attention away from discrete pain stimuli to include physical, cognitive, and affective therapeutic targets [7]. There is growing recognition of the potential benefits of VR for chronic pain [7,8,9,10,11], but our understanding of the mechanisms of its effect is just emerging, and pediatric studies are still rare. Trost et al. [12,13] pioneered the use of VR to deliver graded-exposure therapy targeting pain-related fear and disability in adults with chronic pain, recognizing VR’s power to facilitate pain-related movement in the presence of fear and behavioral avoidance. VR also has the potential to enhance motivation and engagement in physical rehabilitation and to incorporate real-time feedback for both clinicians and patients. Furthermore, evidence drawn from studies of VR in neuro-rehabilitation [14,15] supports the ability of VR interventions to facilitate neuronal changes that promote learning and recovery.

To date, the application of VR in pediatric chronic pain rehabilitation has been largely limited to the physical therapy context, with increasing movement being the main treatment target [16,17]. VR has untapped potential to address more holistic, integrative functional goals such as the return to school, a goal that crosses the disciplinary boundaries of physical, occupational, and psychological treatments. Given the increasing accessibility of VR interventions through declining costs and growing portability and user-friendliness, their particular fit for pediatric populations, and their ability to complement and enhance standard approaches [18], this is an important and timely area of innovation in our field.

School functioning is a major challenge for youths with chronic pain and is a central treatment goal of pediatric pain treatment. Youths with chronic pain report significantly more chronic school absences (defined by the US Department of Education as ≥11 missed school days within 12 months [19]) compared to youths without chronic pain, and childhood pain in the US has been associated with 22.2 million missed school days. In addition to struggles with school attendance, our own and others’ studies demonstrate declines in academic performance and an impaired ability to cope with classroom and social demands among youths with chronic pain [20,21,22,23]. 

Our prior research shows that fear of pain, pain catastrophizing, and behavioral avoidance are major drivers of school impairment in this population [19,24]. Treatment of pediatric chronic pain and disability must therefore include efforts to address school impairment from both physical and psychological perspectives in order to foster the successful achievement of academic, social, and other developmental goals and to prevent the emergence of longstanding patterns of school refusal or avoidance. Among many children and adolescents with pain, school avoidance becomes significant enough to be characterized as a school phobia [25,26,27]. As with other phobias, it is often best treated through a graded-exposure approach, an approach that has been shown to be effectively enhanced with VR. 

To leverage the benefits of VR while meeting the need to improve school functioning in youths with chronic pain, we set out to develop a VR-based school simulation experience incorporating both the physical and psychological challenges associated with returning to school following participation in intensive interdisciplinary pain treatment (IIPT), the current gold standard in treatment for complex pediatric chronic pain [28]. In 2019, an international working group published consensus-based recommendations and methodological best practices for VR studies in health care [29]. Adapting the FDA’s three-phase drug development model, VR1 studies are those in which researchers use human-centered design principles to develop software collaboratively with patients and clinicians; VR2 studies evaluate software usability and acceptance and potential adverse effects in clinical practice; VR3 studies are randomized controlled efficacy trials of VR interventions. The working group emphasized the crucial importance of including the patients’ perspectives throughout the development of VR treatments and the need to thoroughly collect and integrate patients’ feedback through participatory research, user-centered design thinking, and multidisciplinary collaboration [29]. Thus, our efforts began with a VR1 study with a focus on user input from both patients and clinicians to help to shape our ultimate intervention. 

We adopted a two-phased approach to a VR1-level study. In phase I, we sought to employ a focus group methodology to identify salient physical and psychological barriers to school functioning as identified by youths with chronic pain and to inform content to be included within a VR school intervention. We worked closely with our software development team at the MIT Nano Immersion lab to incorporate this feedback directly into software design. In phase II, we conducted a pilot test of a beta version of the “vReal-School” intervention and collected both quantitative and qualitative feedback from both patient and clinician users in the context of pediatric IIPT treatment. Together, these efforts yielded valuable information to aid in the development of a user-centered, immersive, and engaging simulation to foster improved school functioning in youths with chronic pain. 

## 2. Materials and Methods

The study was approved by the Boston Children’s Hospital institutional review board prior to the onset of any study activities. 

### 2.1. Phase I—Content Focus Groups

We conducted four focus groups each consisting of 4–5 current or recent pediatric IIPT patients. The final numbers of participants and groups were based on the criteria of thematic saturation of the focus group content. Each focus group was 45–60 min in duration. Groups took place between October 2021 and February 2022 and were conducted remotely via the secure, HIPAA-compliant Zoom video platform in consideration of the COVID-19 pandemic. Groups were led by the study investigator (DL) who also oversees psychological services in the IIPT program. A second member of the study team (KK or LM) also participated in order to take notes and facilitate the recording of the sessions. Groups were audio-recorded, transcribed via a professional hospital-approved transcription service, and analyzed using Dedoose 9.0 [30], a qualitative analysis software, to highlight prominent themes and inform the development of relevant scenarios for our VR simulation. 

Materials: A semi-structured group interview guide was developed to focus the discussions. This guide included questions designed to solicit participants’ views on aspects of school functioning that they view as particularly challenging to navigate with chronic pain. Questions sought to elicit both physical obstacles related to youths’ pain symptoms (e.g., challenges with physically navigating the school building, sitting for extended periods, walking through crowded hallways) as well as emotional challenges (e.g., worry about teachers not believing their symptoms being real, being teased or bullied by peers, feeling anxious about the ability to do schoolwork). The guide also prompted participants to identify any alleviating factors that help them to feel more successful in the school setting, and their views regarding how well the current IIPT treatment addresses school challenges effectively. (The interview guide is available from the authors by request.)

Participants: Focus group participants were current or recent IIPT patients who were enrolled in middle or high school and could communicate sufficiently well to participate in groups led in English. We sought to recruit groups whose characteristics reflected the pediatric IIPT patient population (i.e., predominantly female with a range of presenting pain issues). Given the virtual nature of the groups, geographic location was not a limiting factor, even if patients had completed the program and returned home. Participants received $20 Amazon gift cards as tokens of appreciation for their time and insights. 

### 2.2. Phase II—Pilot Testing and User Feedback

The goal of the pilot testing phase was to obtain feedback on the feasibility and acceptability of the VR simulation in the IIPT setting, identify any hurdles to implementation, and gather preliminary feedback on levels of immersion and engagement in this type of intervention. Given the aim of developing a useful clinical tool, perspectives of both patients and clinicians were incorporated. 

Before having patients engage with vRS, clinicians at the IIPT tested out the intervention and provided feedback. We then recruited and consented current IIPT patients to participate in pilot testing. Study treatment was a one-session exposure to the vReal-School simulation. Sessions were no more than 30 min in duration and were fully supervised in the IIPT environment by the PI, an IIPT clinician, and a technical expert. A research assistant was also on site to provide additional support as needed. 

Materials: A prototype version of the vReal-School intervention was developed by our study team engineering collaborators at the MIT.nano Immersion Laboratory. The core hardware system for the prototype was the HP Omnicept G2 VR headset, equipped with heart rate monitoring, eye tracking, pupil dilation measurement, and facial expression analysis capabilities. This headset offers an immersive visual experience while providing real-time insights into participants’ physiological responses. Additionally, the study incorporates in-simulation event timers strategically placed within the VR scenarios to induce controlled environmental changes, aiding in the observation of participants’ reactions and behaviors. These timers are synchronized with the headset’s physiological and eye tracking data, enabling the accurate correlation of responses with specific VR events. Together, these integrated materials enabled an in-depth exploration of user experiences, cognitive engagement, and physiological reactions in virtual environments in a full-scale intervention trial. These physiological monitoring tools were included in the current pilot test for the purposes of establishing their feasibility for inclusion in future trials of vRS. 

Participants: Pilot test participants were a convenience sample of current IIPT patients who were enrolled in middle or high school. Patients are accepted into IIPT on the basis of chronic pain conditions that have been refractory to standard outpatient treatments and have been well-characterized in other studies [31,32]. 

Measures:

1. Post-VR session open-ended patient interview. We adapted a brief, open-ended interview developed by Griffin et al. [33] to collect feedback on the VR experience from patients. Questions included prompts such as “Tell me about what happened when you were in VR”, “Tell me about the parts that you expected”, “Tell me about the parts that you did NOT expect”, and “If you could change anything about this, how would you make it better?”
2. Child Presence Questionnaire: To measure the immersiveness of the pilot intervention, we adapted the Child Presence Questionnaire [34,35]) to obtain participant feedback on their level of engagement with the VR. Questions on this measure included “Did you feel like you were in control of the [VR experience]?”, “Did you feel like you were really there?”, “Were you interested in what you saw?”, “Did the way things moved look real?” Responses were rated on a three-point scale including “No”, “A little”, or “A lot”. This measure has been recommended for the evaluation of pediatric VR interventions [7].
3. Post-VR session clinician feedback: We also obtained open-ended feedback from IIPT clinical staff to assess any perceived impacts of the VR intervention on clinical workflow in this setting and how clinicians envision using this tool in their clinical care.

## 3. Results

### 3.1. Summary of Phase I Findings

We completed four focus groups with a total of 19 participants (fifteen current IIPT patients, four recent program graduates). The majority (78.9%) of the participants identified as female, 15.8% as male, and 5.3% as non-binary. The mean age of participants was 15.11 years. Participants were White (84.2%) and Asian (10.5%) (one participant did not report their race/ethnicity). Participants reported an average of 17.8 months since their initial onset of their chronic pain symptoms. A total of 52.6% of participants had a diagnosis of musculoskeletal pain; 26.3% of participants had headaches; 15.8% of participants had abdominal pain; and 5.3% of participants were diagnosed with Complex Regional Pain Syndrome. 

Two raters (DL, KK) utilized a thematic analysis and an inductive coding approach [36,37]. The thematic analysis was undertaken with the goal of informing the development of the VR school simulations, so data coding was focused on identifying the themes and experiences that were most informative to our VR development process. The two coders brought different backgrounds to the qualitative analysis process—one (DL) had two decades of clinical experience in pediatric pain treatment and the other (KK) came to the project with previous qualitative research training but less familiarity with the clinical population. This yielded four main themes from the data. Subthemes were identified under each of these overarching themes. Coding discrepancies between raters were resolved through a discussion-based consensus process. The following themes and subthemes were captured to represent key challenges faced by youths with pain in school and potential strategies for navigating these challenges:

1. Physical/Environmental Challenges and Solutions: Challenges related to navigating the physical spaces and elements of school, solutions for doing this, or pain-coping strategies that involve physical movement (note: under this category, participants frequently referenced experiences with remote school during the pandemic. Given our goal of creating an intervention to facilitate success in in-person school environments, we did not analyze responses focused exclusively on remote learning). Environmental challenges included aspects of the built school environment that exacerbate pain, e.g., screens, noise, and hard chairs. An example statement is “*It’s just like overwhelming, just all the noise and people talking.*” Suggested solutions include physical coping methods, i.e., movement-based strategies for coping with pain. An example statement is “*Learning to use any downtime in school effectively, even if that is just your teacher gives you five minutes to go in the hallway and stretch has definitely been one of the greatest things for me.*”

2. Academic Challenges and Solutions included challenges related to the work in school or solutions for managing these. Under this theme, challenges that appeared the most among participants included limited attention/concentration due to pain. Example statements are “*I couldn’t think. Like I couldn’t think while I was in so much pain*” and “*It was hard to focus on like doing school or studying when I was in pain. I mean I’m always in pain, so it was kind of hard to like be able to like sit down and really focus on getting work and being productive when I was always distracted by that.*” Another challenge in this category was related to strategic time management. Participants generally concurred that learning to manage time effectively while coping with pain is a key skill to develop. They offered examples of solutions, such as “*Figuring out like when I can let myself rest and when I should be doing other things…all of that productivity*” and “*Using any downtime in school effectively, even if that is just your teacher gives you five minutes to go in the hallway and stretch has definitely been one of the greatest things for me and just making schedules and figuring out how I’m going to spend my time is very helpful because then I don’t end up getting distracted and doing something useless for eight hours.*”

Within this theme of academic challenges and solutions, participants also described how the IIPT program currently tries to work on these issues and how these efforts may, at times, be limited by being in a clinical environment. An example statement is “*Towards the end of my time at the [IIPT], they did like a couple of weeks where I mostly did like all schoolwork, so it was like three or four hours like chunked together like school where I was like sitting there doing stuff, and then every hour, I’d put my backpack on, and we’d like go for a walk to simulate like transitioning the hallway, so that was kind of nice like to see if I could like do the work, but I’d definitely say that it didn’t really simulate school because it was just me sitting in a room by myself instead of like sitting in the classroom. I think they did the best they could with what they had.*” Another participant noted ways in which the clinical setting lacks physical elements of an actual school setting: “*I think for people who are in middle school and up it would help if you could switch rooms or like go for like a little walk, pretend that you’re going to like a locker or something and then go back. I don’t know, something like that to make a bit more realistic.*” In addition, participants described the importance of having support from the treatment team to manage school re-entry. An example statement is “*I have a lot of worries about school and school definitely brings a lot of pressure and extra pain... So, I think that like trying to talk about school with the staff here and deal with it, I think is helpful for me.*”

3. Peer Interaction Challenges and Solutions: Participants touched on various issues related to interacting with peers in the school setting. The most commonly coded subtheme within this theme was feeling different from or being mistreated by peers. Example statements are “*Yeah, I mean I definitely think the social stuff was the hardest, and part of it there just isn’t a great way to prepare with being around kids your own age like in a classroom setting without actually like doing it*” and “*I’m a bit afraid that I might get bullied for it [my pain] because there’s some not-nice kids in my school.*” A related subtheme focused on feeling disbelieved by peers or having to justify themselves. Example statements are “*A lot of people make assumptions about like why you were gone, like start rumors, and like just like when coming back, it’s kind of like a shock to people sometimes*” and “*I think like people just don’t really understand it very well, and so trying to use accommodations can definitely be socially awkward, and that’s definitely a challenge, like people really just don’t understand and you have to find ways to explain,*” 

Focus group participants also described trying to hide pain from peers or strategically redirecting conversations away from pain. An example statement is “*I don’t know if I would rather people know about my chronic pain or not know just because it’s like people talking and like I want people to know how hard I’m working almost, but then it’s not easy for me to talk about it, and I don’t really want to talk about it.*” Participants also highlighted some positive peer interactions around pain or solutions for fostering peer interactions that are not focused on pain. Example statements are “*Maybe you have like a friend tell you what you missed for those like, I don’t know, 15 min or 10 min that you were gone* [to use strategies to cope with pain] *so that you can just easily catch up during a free period*” and “*Usually people like to talk about themselves, and if you redirect the conversation to something like that happened over the weekend, that kind of works.*”

4. Teacher Interaction Challenges and Solutions: The final theme that was identified from our patient focus groups emphasized interactions with teachers in the school setting. Within this theme, many comments focused on youths with pain feeling disbelieved or misunderstood by teachers. An example statement is “*You know, some of the nurses at my school, it’s like one said, ‘I’ve seen this before. It’s just the stress of being here and just wanting to go home to play video games,’ which is not true at all*.” Some participants offered potential solutions to address this challenge, emphasizing the benefits of improving communication. An example statement is “*I think just working on like methods of communicating with teachers, I don’t know, like about the things that are more difficult. Like it’s hard to talk about having missed school, but it’s really hard to talk about when the teacher is actively keeping you from being able to like use your 504-plan for something when there’s a problem. I think that is something that even in small ways probably most of us will run into, and just talking out ways to deal with that and stuff with that could be very, very helpful.*”

Advocating for oneself was another subtheme within the theme of teacher interactions. This included both challenges around the need to advocate in order to obtain what is needed as well as success stories regarding successful self-advocacy. Example statements are “*I think I have less confidence in self-advocating because I think it’ll make me feel weak, and I don’t really want to admit vulnerability to people who I don’t know so well. And like being back at school and being like I need a break, just feels so like*—*it just seems silly, but I think that*—*I’m working on that*” and “*We talked with some of my teachers and guidance counselors about what I would have to do and came up with a revised 504-plan together and then like practiced what I would have to do through the day.*” Participants also identified positive teacher interactions, ways in which they worked together positively with teachers to maximize success with coping with pain at school. An example statement is “*I made a secret code with my teachers where I’d just put down a sticky note on my desk, and they let me leave the classroom without actually signaling to any other kids.*”

The user-centered themes highlighted in the focus groups directly informed the content included in the current vRS prototype [38]. In addition to the open-ended discussion of challenges and solutions to functioning with pain in the school setting, the interviewer also asked patient participants to reflect on the concept of a VR-based school simulation in the context of the treatment program. Several useful specific suggestions were offered. Examples of suggestions that were the most directly incorporated into the pilot simulation included the following:

1. Make sure that the VR simulation is appropriate for the grade level. There should be high-school and middle-school versions of the simulation.
2. If the participant has to take a break from the classroom, incorporate realistic stressors—having to catch up on the content when you come back, having kids stare at you, and teachers being reluctant to provide breaks.
3. Make it look, sound, and feel like a real school—walls and lockers should be covered in posters, flyers, stickers, etc. There should be things on the floor like crumpled papers and pen caps. Hallways must be noisy and chaotic. Incorporate practices of sitting on hard chairs.
4. Include environments like the cafeteria, the auditorium for assemblies, and gym classes.
5. Tailor the challenges to different pain conditions—for example, a student with headaches may find the fluorescent lights challenging, while someone with leg pain might be more worried about getting bumped into in the hallway.
6. Incorporate mini games and rewards to make it more motivating.

### 3.2. Summary of Phase II Findings

Phase II was a pilot test of our vRS prototype in our BCH IIPT setting and demonstrates our successful collaboration with the MIT Immersion Lab in the collaborative design, implementation, and evaluation of a VR trial within the clinical population. 

Five clinicians (two physical therapists, one occupational therapist, one social worker) tested the intervention prior to the patient pilot. Some helpful changes that we integrated prior to presenting the prototype to patients were as follows:

1. The majority of clinicians (80%) reported some level of nausea or dizziness after going through the VR and most expressed concern that the IIPT patient population could be especially vulnerable to this effect. This was addressed through the addition of increased grounding cues to provide users with a more stable sense of spatial orientation. For the patient pilot, we also transitioned to the use of higher-end GPU hardware (HP Omnicept G2) with eye calibration and IPD (interpupillary distance) tailored to individual users to enhance the accuracy of gaze tracking, increase the smoothness of the visual feedback, and reduce discomfort.
2. The environment needed to be more interactive to feel like a real school, (e.g., noisier, more people in the hallways/bumping into you). We added more stimuli in the hallways and also increased the object permanency so that if the user ran into another person or an object, they would receive haptic feedback.
3. To address the varying levels of VR familiarity, we added a demonstration portion at the start of the intervention where we provided basic navigation instructions. Participants were then allowed to practice these navigations until they felt like they had gained control over the character and were comfortable with moving into the actual school simulation.
4. To clarify the goals of the experience, we added instructions as well as a checklist of tasks that the user had to complete. Users could access this list and instructions anytime throughout the simulation if they needed guidance.

Once these changes were integrated, patients engaged with vRS in the clinical setting. We obtained feedback from both patients (*n* = 9) and their treating clinicians (*n* = eleven; nine PTs, six OTs, two behavioral clinicians) via a combination of semi-structured interviews and validated self-report measures. The mean age of patient participants was 14.37 years, and 66.7% of participants identified as female and 33.3% identified as male. All participants identified as White. The average duration of pain was 30.9 months. The primary pain diagnoses of participants included headaches (55.6%), CRPS (22.2%), musculoskeletal pain (11.1%), and abdominal pain (11.1%). 

The results indicate high levels of patient engagement/immersion, as indicated by a mean total score of 17.0 (score range 0–22) on the Child Presence Measure (CPM). This score exceeds published means indicating high levels of immersion [35]. Scores were consistently high across CPM subscales of realism, transportation, and involvement/immersion.

Table 1, Table 2 and Table 3 report feedback from patient and clinician users during post-pilot interviews. Authors approached these open-ended interviews with a similar approach to the inductive thematic analysis, as described for the phase I focus group data. Participants were prompted to give feedback about the VR experience (both positive and constructive) and to describe whether and how they felt this tool could be used in the context of intensive pain rehabilitation.

Regarding the positive aspects of vRS, many participants experienced the simulation as realistic and easy to use. They valued the exposure to both physical and psychological challenges that they might face in the school environment, and they highlighted ways in which vRS allowed them to address these challenges in ways that are not otherwise available in the IIPT setting (see Table 1 for more details on the participant feedback provided).

**Table 1:** vRS Pilot Stakeholder Feedback—Positive Feedback Illustrative Quotes.

Positive Feedback	Patient Quotes	Clinician Quotes
Realistic	“What did feel real was the bells and the talking and all that. That’s what my school looked like.” “It’s a lot more real-like, instead of being in a hospital setting, you’re actually in a school setting.”	“The noise and scenes, especially how kids need to figure out the class schedule and where to go, kids moving around in hallways, noise was realistic.”
Easy to use	“I felt like it was pretty easy and I don’t play a ton of video games or anything, I think I was doing the control a little wrong and having a little bit of a hard time but it wasn’t super complicated or anything it wasn’t like laggy which I feel like sometimes games that are taking input from more than just the controller sometimes do that.”	
Exposure to physical and psychological triggers	“Having that weight (of VR equipment backpack) is definitely helpful.” “I think this could help to simulate those stressful situations you might find yourself in when you do return to school, like where people ask you questions.”	“Wearing the backpack and engaging in school tasks makes it a very helpful tool for both physical and social treatment goals.” “(I can see myself using it) as a means of engaging in auditory/visual desensitization”
Addresses goals that can’t otherwise be easily achieved in clinic	“There was a bit of confusion that kind of adds to the realistic school experience of like not knowing where to go. I feel like that might help get us used to regular school.”	Offers a way to practice navigating a school setting with the pressures that can’t be simulated in clinic This would be helpful as a transition to the school environment to practice strategies and gain exposure to potentially triggering contexts in a controlled manner

Regarding constructive feedback, participants expressed dissatisfaction with the current teleportation approach to navigating the environment. Incorporating actual physical navigation while in vRS is a goal for the next iteration but was limited by space constraints in the test setting. Some participants noted shortcomings to the level of realism. Some participants suggested more interactive capabilities, also planned for subsequent iterations of vRS. The majority of constructive feedback comments focused on incorporating additional features, such as different scenarios and tasks to complete, and the ability to increase the challenges that users face in the simulation, such as incorporating stressful social interactions, time pressures to navigate the hallways, etc. (see Table 2).

**Table 2:** vRS Pilot Stakeholder Feedback—Areas for Improvement Illustrative Quotes.

Areas for Improvement	Patient Quotes	Clinician Quotes
Movement	“Fix the teleportation, it’s kind of wonky.” “For me it would be easier to like run around in the simulated environment because I am uncomfortable with my actual school environment.” “I think really the biggest key piece that was missing was that like smooth movement of walking.”	“Require actual physical navigation vs. teleportation.”
Problems with Realism	“Needs to be more chaotic.” “Too cartoony.”	“Some of the portals gave away the answer on where to go (for example: finding the right locker has the portal in front of it).”
Interactivity	“Make it more interactive, able to talk to people.”	“Make the people in the VR more realistic looking, more interactive (ask questions!).”
More tasks and scenarios	“I think it would be kind of cool to have like a more like you have to grab multiple things and put it in your backpack or something on your desk or under your desk like something along those lines.”	“Add more settings—(cafeteria, playground, gym), more people, noises, complex tasks to complete.”
Increase stress of the demands	“I think it would be helpful to have that, being able to have people actually talking in the background and you’re contributing or doing something that might bother someone. Or if people move or come near you, or something like that, that would make more feelings, because those things happen to me and that was really stressful.”	“The stressors of social anxiety or pressure of getting feedback on an assignment or not getting a perfect grade aren’t there yet.” “Add more time pressure/deadlines to physically get through hallways, etc.”

Finally, users provided reflections on the potential clinical utility of vRS in the pain rehabilitation setting. Consistent with the developers’ visions for this clinical tool, users highlighted the benefits of exposure to stress and pain triggers, both physical and emotional, in the school setting and the opportunity to develop and employ coping strategies in a realistic environment. Clinicians did raise concerns regarding the limitations of the program’s use, such as space constraints that could limit the ability to move around as if in a school building, as well as the need to titrate exposure based on an individual patient’s tolerance. Overall, both patients and clinicians saw the potential for vRS to serve as a useful tool that could be employed in different ways across disciplines in the context of interdisciplinary pain treatment. See Table 3.

**Table 3:** vRS Pilot Stakeholder Feedback—Treatment Relevance Illustrative Quotes.

Treatment Relevance	Patient Quotes	Clinician Quotes
Exposure to physical triggers	“It’s a really helpful way to get used to noises, lights, things that are hard about school with headaches.” “It definitely would help with desensitization. People might need to get used to different noises coming from places, like chit-chatting people, or like the bell ringing in the school.”	“I would use it as a means of engaging in auditory/visual desensitization, as a distraction to support improved mobility/standing, as a way of overcoming some school anxiety.”
Working through emotional challenges	“I think it also could be helpful in (…) simulating like, if people were to ask you questions when you came back to school. Like, having little characters be like ‘Hey, where were you these past couple of weeks?’ Because I feel like that’s something a lot of people dread about going back to school after this program: what people are going to ask them when they come back.”	
Opportunity to develop coping strategies	“It would help me a lot with coping strategies before I go back to school that I can fall back on in a school setting.”	“Would be most helpful after the patient has learned and practiced strategies to use at school within IIPT.”
Need to attend to space constraints and patient tolerance		“Important to consider safety awareness (e.g., prone to dizziness), physical tolerance.” “Make sure the physical environment is set up to support this—patients will need room to physically move through the experience.”
Potential to be used across multiple disciplines to address various school re-entry challenges	“I have missed a lot of school so it would be very helpful for me because I haven’t been to a full day of school in a while. Even if you simulate for an hour that’s really helpful with the worry over what it’s like to go back.”	“I could see OT and psych using this more within the current simulation, but if there were more tasks related to mobility/gym class settings, it is something I (PT) would also use to help them return to school.”

## 4. Discussion

The current study describes the initial phases of a user-centered, design-thinking based process to develop an engaging immersive technology intervention using virtual reality to promote school functioning in youths with chronic pain, with applications for treatment providers across multiple disciplines working with patients on school functioning goals. In these early stages of designing vReal-School, we emphasize the need to ground our intervention firmly in the patient experience and to obtain and incorporate feedback from both the patients and clinicians who would ultimately benefit from this digital health tool. Through focus groups and pilot testing we elicited invaluable feedback to guide the subsequent stages of intervention development.

We began this process with patient focus groups to understand patients’ current experiences with coping with pain in the school setting, how our current treatment approaches helped with this, and what gaps remain in achieving this goal that could potentially be filled by our planned intervention. Our thematic analysis of patients’ perspectives on coping with pain in the school setting identified four major themes capturing different types of challenges and goals related to coping with pain in the school setting—physical environment challenges and solutions, academic challenges and solutions, peer interaction challenges and supports, and teacher interaction challenges and supports. These findings are consistent with previous research on the obstacles that youths with chronic pain identify as salient in the school setting [27]. As part of a user-centered approach to designing a clinically useful VR-based intervention, we began our development process with this stakeholder focus group approach to assure that our intervention is relevant to the challenges faced by youths with chronic pain in the school setting and that it will build upon and enhance some of the current strategies patients have identified as helpful for overcoming these challenges.

In our pilot testing of vRS, we sought the dual perspectives of both patients with chronic pain and clinicians experienced with working with this population on school functioning goals in the IIPT setting. Obtaining both patient and clinician feedback allowed us to understand how this tool might meet the needs of two important stakeholder groups who share treatment goals but might approach these goals differently. Patients and clinicians both identified the strengths of the current simulation and helped to pinpoint goals for future iterative development. Many participants found vRS to offer a realistic, accessible, and immersive school simulation that incorporated some of the challenges that youths with pain find the most overwhelming in the school setting, allowing opportunities for developing ways to cope with and master these challenges. User feedback aligned with our own goals for future vRS iterations, including the incorporation of the ability to physically move around the simulated space, increased interactivity with other avatars in the simulation (both peers and school staff), and the ability to scaffold the level of challenge in the simulation, increasing the demands as the patient builds skills and improves functioning through the course of their IIPT participation. Both patients and clinicians identified several ways in which vRS could help to advance current approaches incorporated in IIPT to address the primary treatment goal of improving school functioning.

The study holds direct relevance to patient care. It reflects the patient voice in elucidating patients’ current challenges associated with functioning with pain in the school setting and how well they perceive their participation in IIPT to equip them with the physical and psychological skills necessary to succeed in that environment following pain treatment. Youths with chronic pain experience numerous challenges in the school setting and endorse working to overcome these challenges as a central goal of their engagement in IIPT. In many ways, IIPTs have found effective approaches to address and treat school impairment in youths with chronic pain. However, there are inherent limitations to providing an exposure-based approach to school impairment in a clinical setting. A more realistic and interactive, digitally enhanced school simulation may advance our ability to successfully reduce school impairment as part of a rehabilitative approach to pediatric pain treatment.

Our vReal-School intervention seeks to incorporate as many identified hurdles to school functioning as possible within its school simulation, so that the experience can be tailored to each patient’s specific areas of challenge and treatment goals and can offer a realistic, impactful way to work through school-based challenges while in the clinical treatment setting. To be useful and effective, this tool needs to incorporate simulations of academic challenges (e.g., sustaining attention in the classroom in spite of pain), physical challenges (e.g., navigating crowded hallways), social challenges with peers (e.g., feeling different from peers, finding ways to engage in social experiences in school in spite of pain), and challenges in interactions with teachers (e.g., feeling disbelieved or dismissed). It also needs to help users to develop effective solutions and coping skills through supported practice, such dealing with unwanted questions from peers, communicating effectively with teachers, and building tolerance for pain triggers such as lights, noise, and crowds that are typical elements of the school experience.

Digital technology interventions, particularly immersive experiences such as VR, represent a novel and powerful approach to engaging patients in a functional–restoration focused approach to pain management. With a growing evidence base supporting the use of these tools to increase physical movement in the context of pain rehabilitation, it is time to expand their use to more complex treatment goals. School functioning is an ideal target for this next frontier in the use of immersive technology in the treatment of pediatric chronic pain, given the importance of both fostering school success as a treatment goal in pediatric pain rehabilitation and the benefits of creating a realistic school environment that patients can experience while receiving the support and guidance of their clinical care team.

The current study is small in scope, but it represents the initial steps in a thorough process of intervention development. The limitations include the small numbers of participants in both the focus groups and pilot testing phases. Although the samples’ demographic homogeneity in terms of race and gender are fairly reflective of the pediatric IIPT patient population, this does limit the study’s potential generalizability. We administered only a single, standardized rating scale in our pilot phase, relying primarily on open-ended feedback from both patients and clinicians. Additional limitations arose from the timing of the study, which overlapped with the height of the COVID-19 pandemic and associated school closures. Many of the participants in both phases of the study had reduced recent experiences with in-person school environments, thereby limiting their perspectives on how the tool could be used in those environments. Given our goal of creating an intervention to facilitate success in in-person school environments, we did not analyze responses that focused exclusively on remote learning.

## 5. Conclusions

Based on the user-centered feedback obtained in this VR1 study, we plan to continue our iterative development of the vRS intervention. The next steps include the integration of mind–body skills coaching, expansion of the collection of integrated physiologic assessments to monitor and shape users’ stress and relaxation responses, and the continued expansion of simulation scenes to provide ample opportunities to tailor the vRS experience to each patient’s individual exposure needs. Our software will continue to evolve. Our engineering partners are researching integrated advanced motion prediction algorithms to synchronize virtual movements with the user’s physical motions, reducing the latency, improving the overall immersion, and allowing for improvements in physical movement as a target of treatment.

We are currently preparing to launch a more extensive, multisite feasibility trial that will include a larger patient sample and a more extensive battery of outcome measurements, following proposed guidelines for the assessment of key domains in trials of VR for chronic pain [39]. As this novel technology and our application of it advances, we will explore modifications of vRS for use in other settings including outpatient clinics and at-home use via interactions with clinicians who can share the VR space virtually. We also hope that the intervention can be modified for populations of youths struggling with school impairments rooted in causes other than chronic pain. Given the envisioned versatility and customizability of the vRS intervention, there is potential for significant impacts of this novel approach to treating school impairment in a wide range of pediatric populations.

## Data Availability

Data are available from the authors on request.
